# Genome-wide identification of the *CPK* gene family and associated responses to calcium stress in *Hemiboea subcapitata*

**DOI:** 10.3389/fpls.2026.1745553

**Published:** 2026-01-28

**Authors:** Tianya Zhang, Yi Dai, Dan Gao, Xiaoguo Xiang, Chunce Guo, Shunbao Lu, Yanjie Zhang

**Affiliations:** 1 College of Life Sciences, Jiangxi Normal University, Nanchang, Jiangxi, China; 2 School of Life Sciences, Key Laboratory of Poyang Lake Environment and Resource Utilization Ministry of Education, Nanchang University, Nanchang, Jiangxi, China; 3 Jiangxi Provincial Key Laboratory of Improved Variety Breeding and Efficient Utilization of Native Tree Species, Forestry College, Jiangxi Agricultural University, Nanchang, Jiangxi, China

**Keywords:** calcium stress, cpk gene family, gene expansion, *Hemiboea subcapitata*, phylogeny

## Abstract

Calcium-dependent protein kinase (CPK) gene family, which can be activated directly by Ca^2+^, plays an important role in Ca^2+^ signal transduction and stress response and is widely present in green plants. So far, the role of *CPK* gene family evolution for species in karst area is far from understanding. *Hemiboea subcapitata* (Gesneriaceae) was used to explore this issue, for its mainly distributing in the karst area of south China. Our results indicated that 32 highly conserved *CPK* genes identified were distributed across 14 chromosomes in *H. subcapitata*. Additionally, 10 gene pairs were generated by fragment replication. Subcellular localization analysis revealed that *HsCPKs* were mainly localized in chloroplasts and cytoplasm. This gene family experienced intron loss events, but its motif structure was highly similar. Phylogenetic analysis showed that the *HsCPKs* were divided into four subfamilies. Subfamilies I and II were under neutral selection, while subfamilies III and IV were under strongly positive selection. The *HsCPKs* showed different expressions in three vegetative organs of *H. subcapitata*. Meanwhile, the expression levels under calcium stress revealed an overall increasing trend for all *HsCPK*s examined. Cis-acting elements analysis revealed that *HsCPKs* contained hormone-responsive elements related to stress. The expansion and evolution of *CPK* gene family in *H. subcapitata* may be related to its adaptation to calcium-rich and stressed habitats. This study provides a valuable understanding for the roles of the *CPK* gene family within karst species.

## 1 Introduction

Calcium-dependent protein kinase (CPK, sometimes abbreviated as CDPK) is a typical Ca^2+^ sensor, which is unique to plants and some protists (Harmon et al., 2000; Harper and Harmon, 2005; Schulz et al., 2013). Researchers speculated that ancestral *CPK* genes may have evolved from the fusion of ancestral *CaMK* genes and the ancestral calmodulin genes (Suen and Choi, 1991; Zhang and Choi, 2001). Currently, 34 *CPK* family members have been identified in the *A. thaliana* (Cheng et al., 2002), and these genes have been confirmed to be involved in signal transduction, stress response, stomatal movement, and other functions (Boudsocq and Sheen, 2013). For instance, Wu et al. (2018) showed that *CPK* gene family participates in Ca^2+^-mediated signaling pathway to regulate key developmental processes in *A. thaliana. CPK21* and *CPK23* were able to phosphorylate the slow-sustained anion channel 1 (SLAC1) of the guard cell in a Ca^2+^-sensitive and Ca^2+^-insensitive manner (Geiger et al., 2010; Shi et al., 2018). In addition, other studies successfully verified that *CPK* genes have been played positive roles in stress response. Such as *GhCDPK4* plays a positive role in the response to drought stress in cotton (Kong et al., 2023). Fan et al. (2023) confirmed that *CPK* expression was significantly up-regulated under calcium deficiency in cultivated peanut (*Arachis hypogaea* L.). Li et al. (2024) demonstrated that *CDPK* signaling pathway in mustard (*Brassica juncea* L.) plays a crucial role in adaptation to cold stress. Therefore, *CPKs* are a significant multi-gene family in various plant species, and they play a crucial role in plant growth, development, and responses to stress.

Karst area is the landform developed on the carbonate rock, resulting in high soil calcium content. In this region, there is poor water retention in the soil layer, leading to drought stress for plant growth and development processes (Ford and Williams, 2007). This unique feature causes vegetation in this area to exhibit characteristics of rock growth, drought tolerance, and preference for calcium-rich environments. In karst regions, high concentrations of calcium ions (Ca²^+^) can act as environmental signals to trigger specific expression of *CPK* genes (Boudsocq and Sheen, 2013). Majority of Gesneriaceae species distributed in Asian karst regions, and many researches have been conducted to explore their tolerance adaptation. For instance, Li et al. (2014) found that *Lysionotus pauciflorus* can tolerate 200 mM high calcium by restricting calcium transport to photosynthetic tissues, and *Boea hygrometrica* only tolerates 20 mM calcium by maintaining protein homeostasis through the *BhDNAJC2* gene. Shi et al. (2022) suggested that the *CPK* gene family in *B. hygrometrica* might respond to drought and salt stress by regulating stomatal movement. The adaptation helps *B. hygrometrica* thrive within arid and water-scarce habitats of karst areas. Mihailova et al. (2023) compared the response of three Gesneriaceae species (*Ramonda serbica*, *Ramonda nathaliae* and *Haberlea rhodopensis*) to cold and freezing stress. The results demonstrated that three species can fully restore their photosynthetic activity through the accumulation of specific protective proteins (dehydrins and ELIPs). Additionally, Legardón and García-Plazaola (2023) found that all known drought-tolerant species belong to the tribe Trichosporeae. These species shared common morphological traits and cope with stress by retaining chlorophyll, enhancing antioxidant systems (polyphenols, ascorbic acid), and accumulating protective proteins (LEA proteins, ELIPs). *Hemiboea subcapitata* (Gesneriaceae), an endemic traditional medicinal plant, is mainly distributed in Asian karst regions (Li and Wang, 2005; Wen et al., 2010). Notably, it inhabits in limestone canyon cliffs and weathered fragmented rock surfaces. Given that the *CPK* gene family plays an important role in Ca^2+^ signal transduction and response to stress in plants (Wei et al., 2014; Atif et al., 2019), we hypothesized that the *CPK* gene family in *H. subcapitata* may have significant implications for its adaptation to karst areas.

To reveal the characteristics of the *CPK* gene family in *H. subcapitata*, we conducted transcriptome sequencing on *H. subcapitata* and identified the *CPK* gene family from the whole genome data. We combined phylogenetic relationships and comparative gene family analysis to explore the evolutionary characteristics of the *CPK* gene family in *H. subcapitata*. We also investigated the expression changes of *CPK* genes under Ca^2+^ stress conditions by RT-qPCR experiments. This research not only uncover the roles of *CPK* genes in the Ca^2+^ stresses of *H. subcapitata*, but also provides valuable genetic resources for the karst adaptation of species.

## 2 Materials and methods

### 2.1 Sampling, RNA sequencing and *de novo* assembly

The living materials of *H. subcapitata* were collected from Guangxi Institute of Botany (Guangxi, China) and cultivated in the laboratory at 26 ± 1°C with a 13:11 h light/dark photoperiod. Fresh roots (2 cm segments of root tips were retained with aged taproots removed), stems (1 cm segments were taken from the middle internodes), and mature functional leaves (petioles were removed) were collected separately from three individuals. Samples of each tissue type were separately placed into labeled cryovial tubes, immediately snap-frozen in liquid nitrogen, then transferred to -80°C for storage, and used for subsequent transcriptome sequencing.

The RNA sequencing was completed in Beijing Novogene Company (Beijing, China). Sequence reads were filtered to eliminate adapter sequences, reads containing more than 5% ambiguous nucleotides, and low-quality reads with a Phred score below 20. The resulting high-quality clean reads were subjected to *de novo* assembly using Trinity software v2.1.1 (Broad Institute of MIT and Harvard, USA). These assembled transcripts were further clustered and processed using Tgicl v2.1 software (MathWorks, Natick, USA) to remove redundant sequences, yielding unigenes. Then, we associated transcriptome gene IDs with CDS Parent attributes and genome sequence names, thereby achieved cross-omics mapping between the transcriptome expression matrix and the genome file.

### 2.2 Analysis of gene expression

The expression levels of *HsCPK* genes were quantified using RNA-Seq by Expectation Maximization (RSEM) and Fragments Per Kilobase of exon per Million fragments mapped (FPKM) (Thiel et al., 2003; Trapnell et al., 2010). Deferentially expressed genes across groups were identified through comparative analysis using the EBSeq software v2 (Langmead and Salzberg, 2012). Unigenes were designated as differentially expressed genes (DEGs) if they met the following statistical thresholds: P-value < 0.05 (Wald test), false discovery rate (FDR) ≤ 0.05, and absolute fold change (FC) ≥ 2. In addition, the transcriptome expression matrix was obtained through expression quantification. The expression matrix was extracted and standardized by Z-Score, where features with absolute Z-scores exceeding the critical threshold of ±1.96 (corresponding to a two-tailed significance level of α=0.05) were considered to exhibit statistically significant expression deviations. The genes with high expression were selected and the expression heatmap was generated in GraphPad prism software v9 (Mitteer and Greer, 2022).

### 2.3 Genome-wide identification of *CPK* gene family in *H. subcapitata*

The genome and amino acid sequences of *H. subcapitata* were downloaded from the China National Center for Bioinformation genome sequence archive (GSA) under accession number GWHFICV00000000 (https://ngdc.cncb.ac.cn/). The blastp program was used to find the *CPK* gene family of *H. subcapitata*, using the protein sequences of the *A. thaliana CPK* gene family as queries (https://www.arabidopsis.org/). Further, we downloaded the Hidden Markov Model (HMM) of the typical structural domain Pkinase and EF hand7 of *CPK* from the Pfam website (https://www.ebi.ac.uk/interpro/), and compared the candidates obtained from BLAST with those retrieved from HMM model. Then, we predicted the protein domains of candidate members of the *CPK* gene family in *H. subcapitata*, and submitted protein sequences to the MEME website (https://meme-suite.org/meme/doc/meme.html) for conservative motif prediction.

### 2.4 Chromosome localization, sub-cellular localization and cis-acting element analysis

Based on the annotation file of the whole genome and the ID number of the *CPK* gene in *H. subcapitata*, the chromosome localization analysis and visualization were studied using the Gene Location Visualize from GTF/GFF tool in TBtools software v2.371 (Chen et al., 2020). Sub-cellular localization prediction of the *CPK* gene family in *H. subcapitata* was performed using WoLF PSORT (https://wolfpsort.hgc.jp/). The 2000 bp sequences upstream of the transcription initiation site of the candidate genes were extracted from *H. subcapitata* the genome sequences. The PlantCARE software (https://bioinformatics.psb.ugent.be/webtools/plantcare/html/) was used to search for cis-acting elements (Lescot et al., 2002).

### 2.5 Sequence alignment and phylogenetic analysis

The Muscle tool (Edger, 2022) in MEGA-X software v5.1 (Kumar et al., 2018) was used to align the protein sequences of the *CPK* gene family members in *H. subcapitata* and *A. thaliana*, with default parameters. Subsequently, we employed the RAxML software v.8.2.12 (Stamatakis, 2014) to reconstruct the Maximum Likelihood (ML) phylogenetic tree. The PROTGAMMAWAG model was selected (Zhong and Betancur, 2017), and 1000 bootstrap replicates were performed.

### 2.6 Analysis of conserved motifs, gene structure, and collinearity

The protein sequences were submitted to the MEME website for conserved motifs prediction. Genome-wide protein sequences were self-aligned using the BLAST tool in TBtools software v2.371 (Chen et al., 2020). Based on the comparison results and the annotation files of the whole genome, gene relationships were obtained utilizing the Quick Run MCScanX Wrapper tool (Wang et al., 2012). The collinearity relationship was visualized using the Circle Gene View tool in TBtools software v2.371 (Chen et al., 2020), based on the annotation files of whole genome and link files of gene relationships.

### 2.7 Select pressure analysis

Nucleotide sequences of the *CPK* gene family in *H. subcapitata* were aligned using the ClustalW tool (Thompson et al., 2003) in MEGA-X software v7 (Kumar et al., 2018) with default values for each parameter; it was converted into.PML format using DAMBE software v7.0.35 (Xia, 2018). Then, the resulting files were used to selection pressure analysis via EasyCodeML software v1.4 (Gao et al., 2019), with the Branch-Site Model selected for the analysis.

### 2.8 RT-qPCR analysis of *CPK* genes

Before Ca^2+^ treatment, the individuals with signs of disease or developmental abnormalities were excluded, and only healthy individuals with consistent growth stages were selected. Twelve healthy individuals were selected and randomly divided into 4 groups, which were treated with CaCl_2_ solutions at concentrations of 0, 5, 10, and 20 mmol/L. The treatment was performed using the root-zone soil surface spraying method, with 50 mL of solution applied each time, once every 2 days. After the five consecutive treatments were completed, the status of root length, stem length, leaf length, leaf width, and plant height were measured to determine the optimal concentration (**Supplementary Table S1**). Subsequently, samples of roots, stems, and leaves from the 0 mmol/L (control) and optimal concentration treatment groups were collected, immediately snap-frozen in liquid nitrogen, and then stored at -80°C for subsequent RNA extraction and quantitative real-time PCR (RT-qPCR).

Total RNA was extracted according to the TianGen RNAprep instructions (Beijing, China). In order to further validate the transcriptome sequencing data, seven *HsCPK* genes were selected for RT-qPCR. The primer sequences used for RT-qPCR were listed in **Supplementary Table S2**. The β-actin was selected as the internal reference gene (Shu et al., 2020; Wang et al., 2017). An RT mix with DNase (All-in-One) (Singabio, China) was used for reverse transcription to remove the contaminated genomic DNA and generate cDNA. RT-qPCR was conducted utilizing the universal SYBR Green qPCR Supermix (Singabio, China). The RT-qPCR cycling conditions included pre-denaturation at 95°C for 120 s, followed by denaturation at 95°C for 5 s, annealing at 60°C for 15 s, extension at 72°C for 30 s, this cycle repeated for 40 cycles. Two biological replicates of the study were performed. The relative expressions of the target genes were calculated using the 2^−ΔΔCT^ method (Livak and Schmittgen, 2001). The data were analyzed by one-way analysis of variance (ANOVA) using the SPSS software v20.0, and the differences were considered statistically significant at *P* < 0.05.

## 3 Results

### 3.1 Basic characteristics and phylogenetic analysis of the *CPK* gene family in *H. subcapitata*

**Figure 1:**
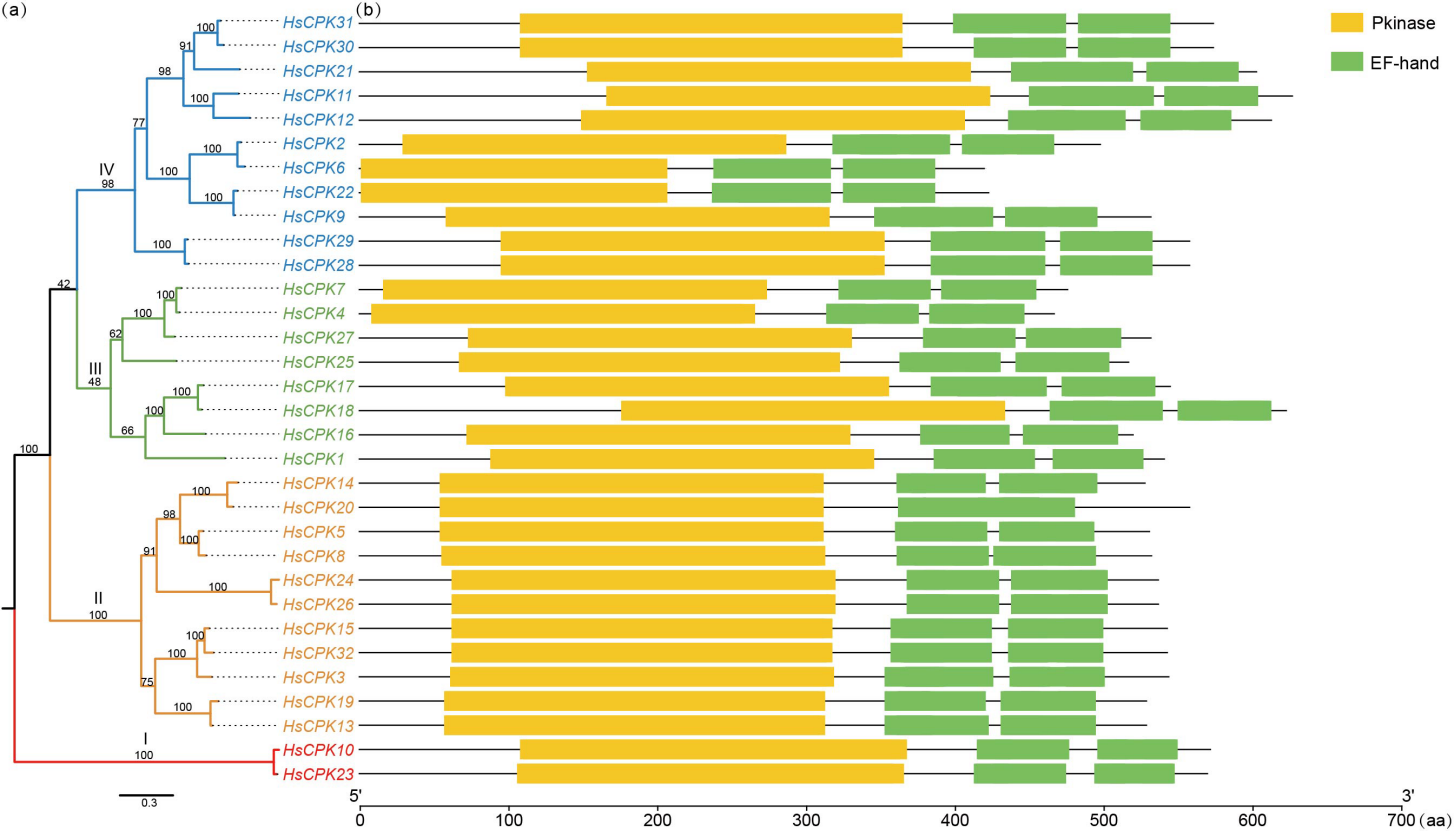
Analysis of the phylogenetic tree and protein domains of the *CPK* gene family. **(a)** Maximum Likelihood (ML) phylogenetic tree of the *CPK* genes in *H*. *subcapitata*. The numbers on the branches indicate the support of each branch. Red represents subfamily I, orange represents subfamily II, green represents subfamily III, and blue represents subfamily IV. **(b)** Protein domains of the *CPK* genes in *H*. *subcapitata*.

By comparing selected conserved domains with the protein sequences of 34 *CPK* genes in *A. thaliana*, there were 32 *CPK* genes identified in *H. subcapitata* (**Figure 1**). These genes were sequentially named *HsCPK1-HsCPK32* based on their positions on the chromosome (**Supplementary Table S3**). These *HsCPK* genes distributed across 14 chromosomes, with the highest number (5) found on chromosomes 1 and 2, and only member on chromosome 5, 10, 12, 13, 14, 16 respectively. Subcellular localization prediction indicated that 14 *HsCPK* genes were localized in chloroplasts, 9 in cytoplasm, 4 in nucleus, 2 in plasma membrane, 2 in mitochondria, and 1 in endoplasmic reticulum. Among them, two genes were shared by chloroplasts and cytoplasm (**Supplementary Table S3**). The maximum likelihood phylogenetic tree showed that *HsCPKs* can be divided into four monophyletic groups. Subfamily I was the first divergent clade, and followed by subfamily II. Subfamily III was sister to subfamily IV (**Figure 1a**). The numbers of *HsCPKs* varied in each subfamily: subfamily I only contained 2 members, subfamilies II and IV contained 11 members respectively, and subfamily III consisted of 8 members.

### 3.2 Conservative motifs and gene structures of *HsCPKs*

**Figure 2:**
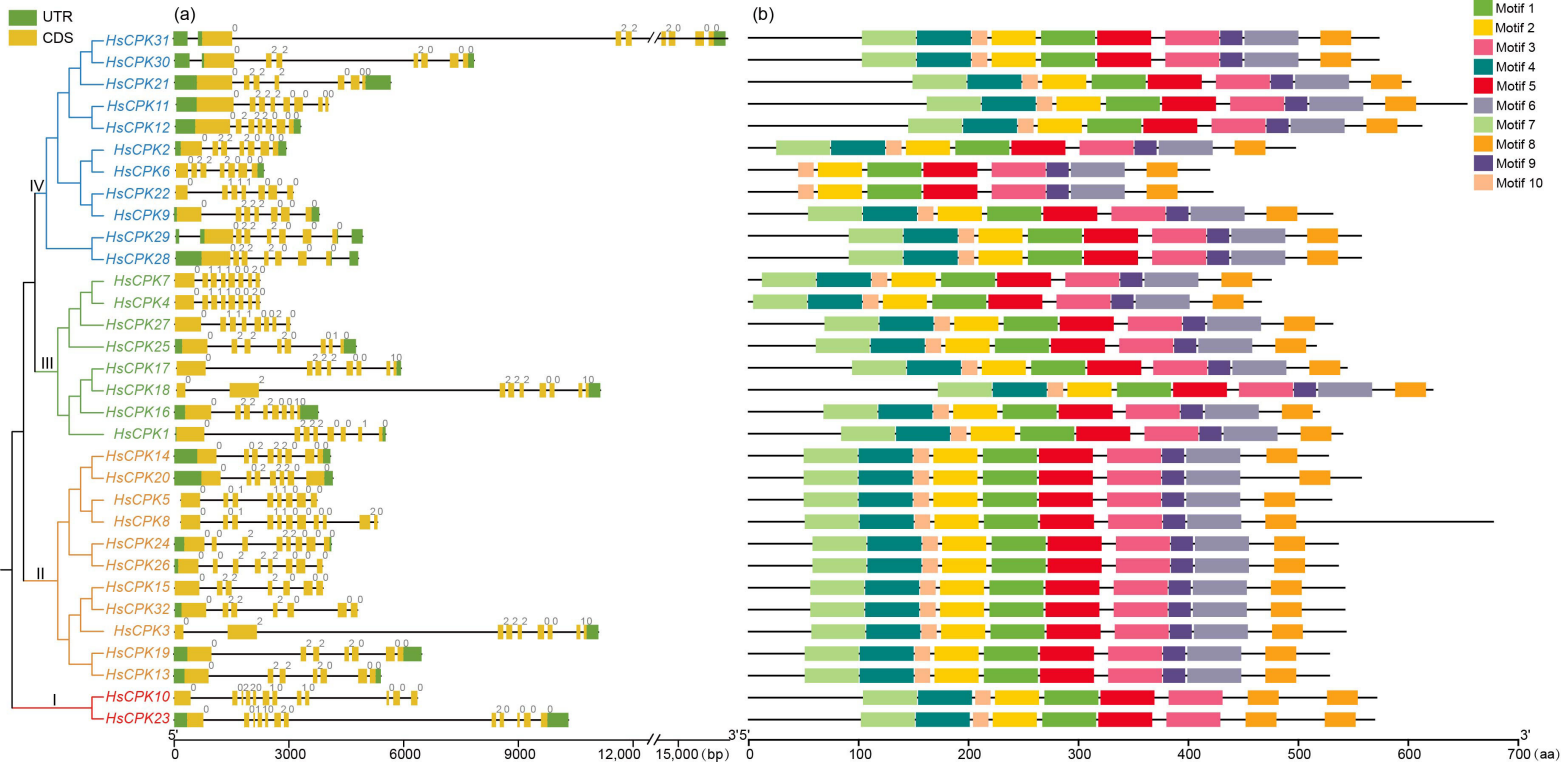
Analysis of the gene structure and motifs structure of the *CPK* gene family. The phylogenetic tree is the same as that in **Figure 1a**. **(a)** The exon-intron structure of the *HsCPK* genes. The lengths of exons and introns for each *HsCPK* gene are shown proportionally. The green boxes, and yellow boxes represent untranslated region (UTR), and coding DNA sequence (CDS), respectively. The numbers represent intron phases. Phase 0: The intron is inserted between two complete codons. Phase 1: The intron is inserted between the first and second bases of a codon. Phase 2: The intron is inserted between the second and third bases of a codon. **(b)** Distribution of conserved motifs in HsCPK proteins. Different conserved motifs are shown in different colored boxes.

The gene structure and motif composition of 32 *HsCPK* genes were shown in **Figure 2**. The results indicated that there were 6–11 introns in the *CPK* gene family members (**Figure 2a**). Subfamily I had the highest number of introns (11-12) with phases of (002201010000) and (00110202000), significantly differing from the other three subfamilies. Subfamily II mainly consists of genes with intron phases of (00222000) and (0222000). The formation of intron phase (00222000) was attributed to the insertion of one intron at the 5’ end for phase (0222000), while *HsCPK20* had a different phase at (0022200) potentially due to loss events at the 3’ end. Additionally, the phase of *HsCPK8* (00111000020) was significantly different from that of *HsCPK5* (00111000) at the 3’ end, indicating potential intron insertion or loss events in these two genes. Seven members in subfamily III have an intron phase of (01110020) or (02220010), with only *HsCPK18* having an intron phase of (022220010), which was possibly due to an insertion event at the 5’ end. In subfamily IV, nine genes have an intron phase of (0222000), the intron phase of *HsCPK11* was (02220000), which may be due to the insertion of an intron at the 3’ end, and *HsCPK22* was (0111000), which may be due to single base insertion or deletion in the exon region.

The 32 *HsCPK* genes contained 8–10 conserved motifs. The 10 distinct motifs were represented by different colors (**Figure 2b**), and the motif sequence were shown in **Supplementary Figure S1**. The motif structure was relatively conserved among gene family members. Of these, 28 genes had an identical motif arrangement, ordered as motif 7, motif 4, motif 10, motif 2, motif 1, motif 5, motif 3, motif 9, motif 6, and motif 8. The remaining 4 genes exhibited minor variations in their motif composition. For instance, the two members of subfamily I (*HsCPK10* and *HsCPK23*) showed consistent motif composition and arrangement, and this subfamily differed significantly from the other three subfamilies, specifically characterized by lacking motif 6 and motif 9 and adding motif 8 at the 3’ end. In subfamily IV, the two members (*HsCPK6* and *HsCPK22*) only contained 8 motifs, with motifs 4 and 7 lacking at the 5’ end.

### 3.3 Collinearity relationship and selection pressure analysis of *HsCPKs*

**Figure 3:**
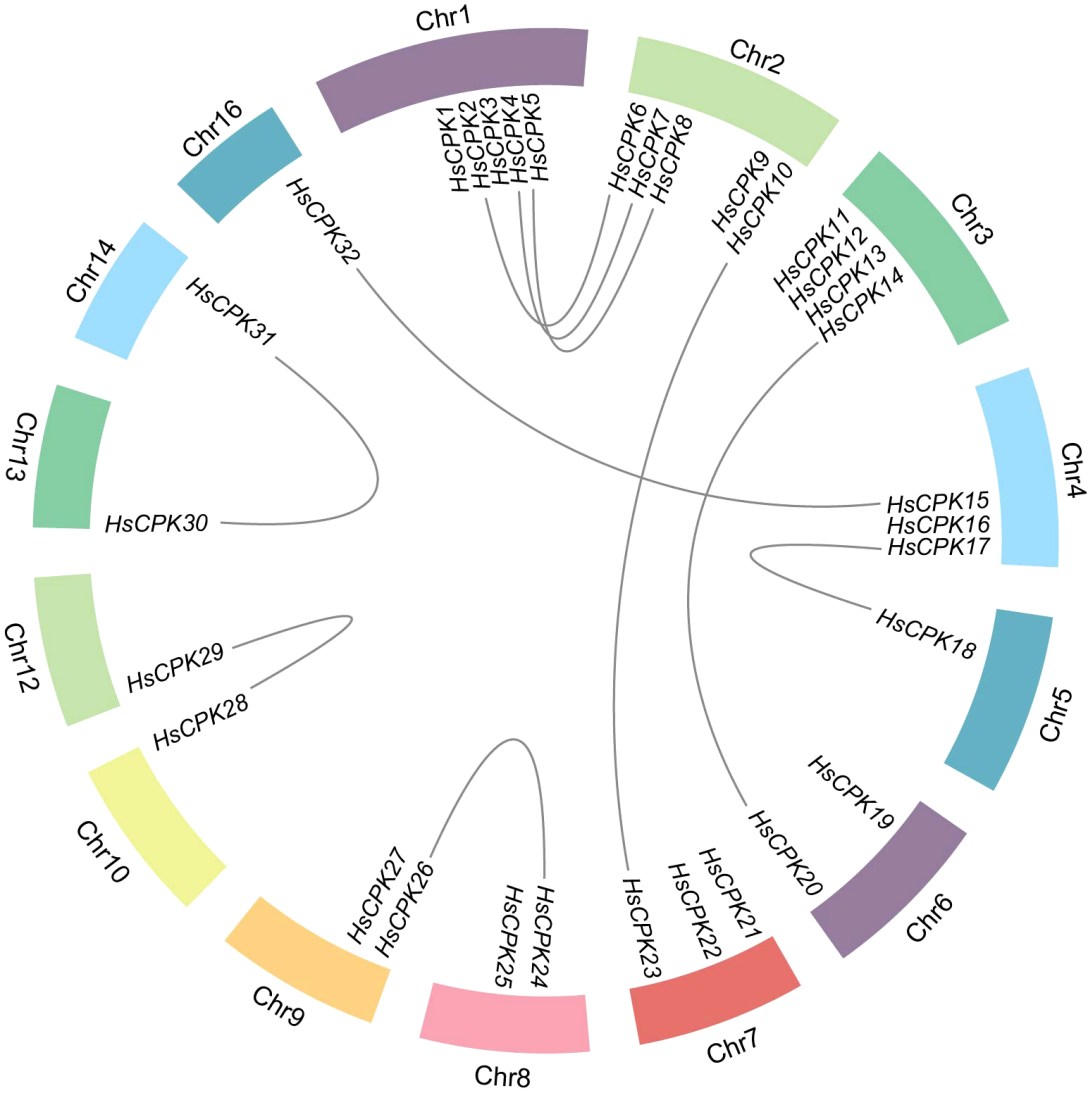
Collinearity of the *CPK* gene family in *H. subcapitata*. Grey lines indicate collinear blocks of *CPK* genes.

The results of collinearity analysis revealed 10 pairs of collinear genes, all generated by segmental duplication (**Figure 3**, **Supplementary Table S4**). These genes pairs were distributed across all four subfamilies: only one pair in subfamily I (*HsCPK10* and *HsCPK23*); four pairs in subfamily II (*HsCPK5* and *HsCPK8*, *HsCPK14* and *HsCPK20*, *HsCPK24* and *HsCPK26*, *HsCPK15* and *HsCPK32*); two pairs in subfamily III (*HsCPK4* and *HsCPK7*, *HsCPK17* and *HsCPK18*); three pairs of genes replicated in subfamily IV (*HsCPK2* and *HsCPK6*, *HsCPK28* and *HsCPK29*, *HsCPK30* and *HsCPK31*).

Selection pressure analysis indicated that subfamilies I (P=0.533190132) and II (P=0.055101493) were subject to neutral selection (P > 0.05). While, subfamily III (P=0.000111608) may also have undergone strongly positive selection, containing 7 positively selected sites, and subfamily IV (P=0.000105812) may have undergone strong positive selection (P<0.01), with 10 positively selected sites.

### 3.4 Cis-acting elements analysis of *HsCPKs*

**Figure 4:**
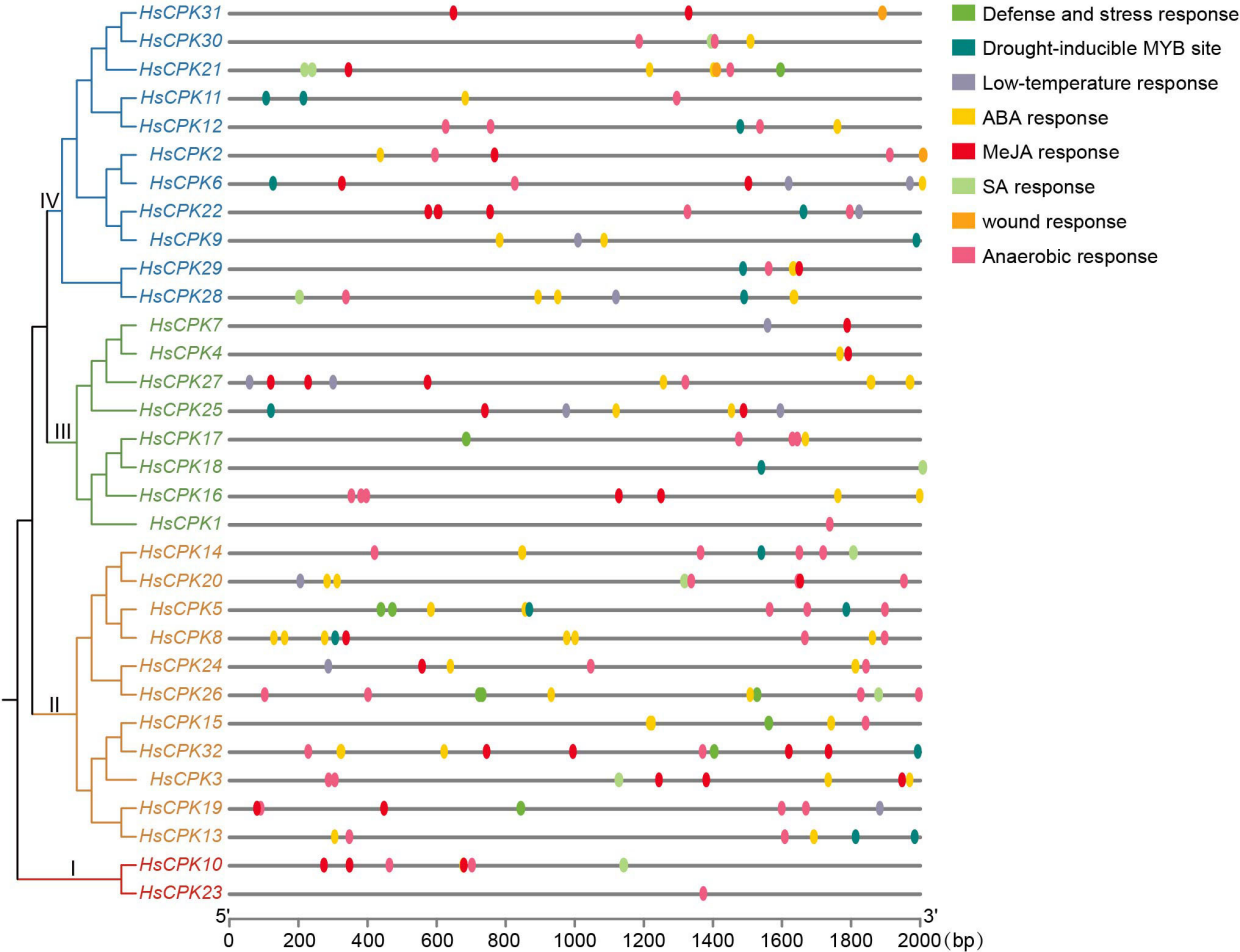
Cis-acting elements analysis of the *CPK* genes in *H. subcapitata*. The phylogenetic tree is the same as that in **Figure 1a**. Defense and stress response: cis-acting element involved in defense and stress responsiveness; ABA response: cis-acting element involved in the abscisic acid responsiveness; Anaerobic response: cis-acting regulatory element essential for the anaerobic induction; Drought-inducible MYB site: MYB binding site involved in drought-inducibility; MeJA response: cis-acting regulatory element involved in the MeJA-responsiveness; Low-temperature response: cis-acting element involved in low-temperature responsiveness; SA response: cis-acting element involved in salicylic acid responsiveness; Wound response: wound-responsive element.

Cis-acting elements analysis in the promoter region (2000 bp upstream) of the *CPK* gene family in *H. subcapitata* revealed that various regulatory elements closely related to stress response (**Figure 4**). The four subfamilies showed functional conservation in element composition. They generally contained MeJA-responsive elements and ABA-responsive elements. This reflects the functional commonality of family members in MeJA and ABA hormone responses.

Meanwhile, significant specific differentiation was observed in the element composition of each subfamily: Subfamily I had a relatively simple element composition, with MeJA-responsive elements and anaerobic-responsive elements as the core. Only *HsCPK10* contained one SA-responsive element. Subfamily II had the most abundant element types, and all genes contained ABA-responsive elements. Compared with other subfamilies, a large number of genes in subfamily II specifically possessed defense and stress response elements (*HsCPK5*, *HsCPK15*, *HsCPK19*, *HsCPK26*, *HsCPK32*). Meanwhile, many genes in subfamily II also contained multiple other elements, including drought-inducible MYB sites (*HsCPK5*, *HsCPK8*, *HsCPK13*, *HsCPK14*, *HsCPK32*) and MeJA-responsive elements (*HsCPK3*, *HsCPK8*, *HsCPK19*, *HsCPK20*, *HsCPK24*, *HsCPK32*). Subfamily III was characterized by the enrichment of low-temperature response elements (*HsCPK7*, *HsCPK25*, *HsCPK27*). This subfamily also widely distributed hormone-responsive (*HsCPK4*, *HsCPK7*, *HsCPK16*, *HsCPK17*, *HsCPK18*, *HsCPK25*, *HsCPK27*) and anaerobic-responsive elements (*HsCPK1*, *HsCPK16*, *HsCPK17*, *HsCPK27*). Subfamily IV specifically possessed wound response elements (*HsCPK2*, *HsCPK21*, *HsCPK29*, *HsCPK31*). Meanwhile, it contained various other elements, including drought-inducible MYB sites (*HsCPK6*, *HsCPK9*, *HsCPK11*, *HsCPK12*, *HsCPK22*, *HsCPK28*, *HsCPK29*) and SA-responsive elements (*HsCPK21*, *HsCPK28*, *HsCPK30*).

### 3.5 The expression levels of the *CPK* gene family

**Figure 5:**
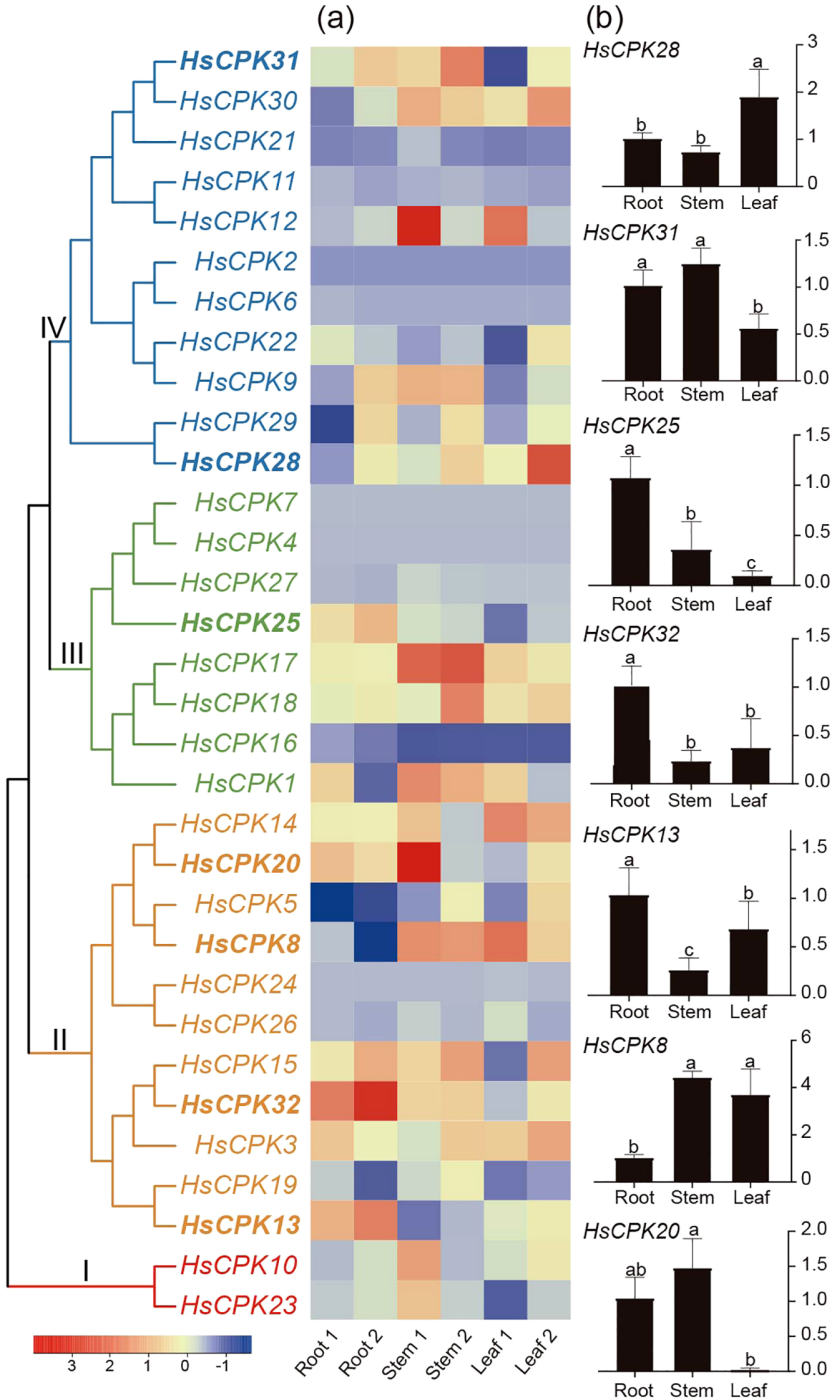
Expression patterns of the *CPK* gene family in different tissues of *H*. *subcapitata*. The phylogenetic tree is the same as that in **Figure 1a**. **(a)** The heatmap of *CPK* genes in three different tissues (root 1, root 2, stem 1, stem 2, leaf 1, and leaf 2); the paired samples for each tissue represent biological replicates. **(b)** Select the expression of *HsCPKs* in different tissues (root, stem and leaf) based on RT-qPCR. Two biological replicates were carried out for each tissue. Different letters indicate a statistically significant difference at *P* < 0.05.

The expression heatmap of the *CPK* gene family in *H. subcapitata* was visualized in **Figure 5a**. Expression analysis showed that *CPK* genes were expressed in three vegetative tissues in *H. subcapitata* (**Figure 5a**). For example, four genes in subfamily II (*HsCPK13*, *HsCPK14*, *HsCPK19*, and *HsCPK32*), three genes in subfamily III (*HsCPK17*, *HsCPK18*, *HsCPK25*), two genes in subfamily IV (*HsCPK29*, *HsCPK31*) were highly expressed in root; three genes in subfamily II (*HsCPK8*, *HsCPK14*, and *HsCPK20*), two genes in subfamily III (*HsCPK17* and *HsCPK18*), and two genes in subfamily IV (*HsCPK12* and *HsCPK31*) were highly expressed in the stem; two genes in subfamily II (*HsCPK8* and *HsCPK14*) and three genes in subfamily IV (*HsCPK12*, *HsCPK28*, *HsCPK30*) were highly expressed in leaf. The expression levels of *HsCPK23* (Subfamily I), *HsCPK5*, *HsCPK15*, *HsCPK19* (subfamily II), and *HsCPK1* (Subfamily III) were relatively low in the three vegetative tissues.

### 3.6 Expression analysis of *CPK* genes based on RT-qPCR

To validate the results presented in the heat map, seven highly expressed genes (*HsCPK8*, *HsCPK13*, *HsCPK20*, *HsCPK25*, *HsCPK28*, *HsCPK31*, *HsCPK32*) were selected from subfamilies II, III and IV for RT-qPCR verification. The results of RT-qPCR indicated expression levels of *HsCPK13*, *HsCPK25* and *HsCPK32* peaked in root, *HsCPK20* exhibited the highest expression in stem, *HsCPK8* and *HsCPK28* showed elevated levels in leaf (**Figure 5b**). Additionally, expression level of *HsCPK31* was higher in root and stem than in leaf, but the differences between root and stem was not significant (**Figure 5b**). These results were consistent with those depicted in the heat map (**Figure 5a**).

**Figure 6:**
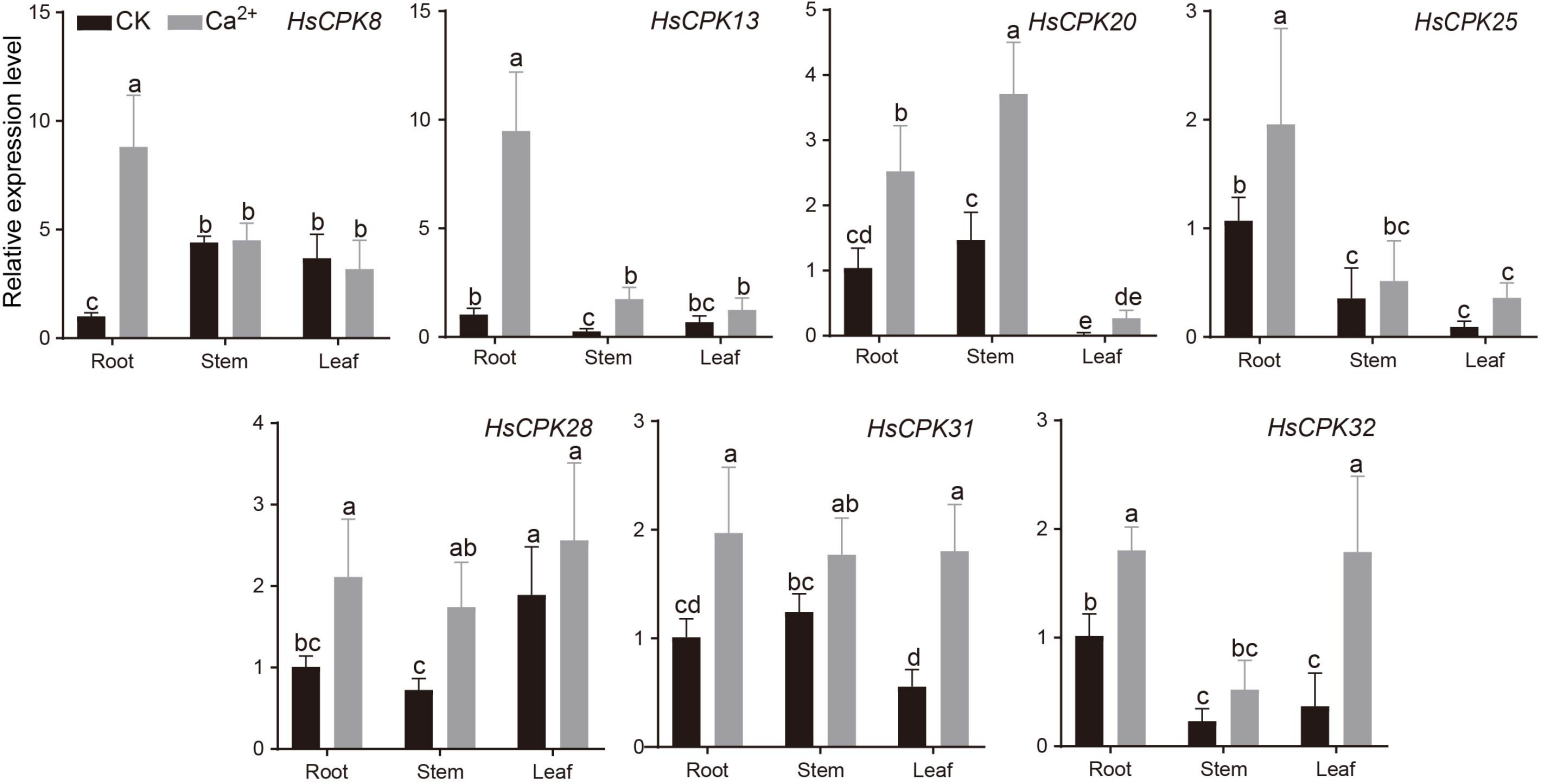
Changes in the relative expression of the *CPK* genes in *H. subcapitata* after calcium stress. Two biological replicates were carried out for each tissue. Different letters indicate a statistically significant difference at *P* < 0.05.

Furthermore, among the different Ca^2+^ concentration treatment experiments (0, 5, 10, 20 mmol/L), the five phytonotic traits (root length, stem length, leaf length, leaf width and plant height) remain stable or increase only at 10 mmol/L Ca^2+^ concentration (**Supplementary Table S1**). Compared with the control group, the expression levels of seven representative genes significantly increased in root following calcium treatment; *HsCPK13*, *HsCPK20*, and *HsCPK28* showed significant increases in stem; while *HsCPK31* and *HsCPK32* exhibited significant increases in leaf (**Figure 6**). Furthermore, following calcium treatment, *HsCPK8, HsCPK13, HsCPK25, HsCPK28, HsCPK31*, and *HsCPK32* exhibited high expression in root; while *HsCPK20, HsCPK28*, and *HsCPK31* showed high expression in stem; *HsCPK28, HsCPK31*, and *HsCPK32* were highly expressed in leaf. Notably, *HsCPK8, HsCPK13*, and *HsCPK25* showed no significant differences in expression between stem and leaf, while *HsCPK28* and *HsCPK31* exhibited no significant expression differences across all three tissues.

## 4 Discussion

### 4.1 Highly conservatism of *CPK* gene family in *H. subcapitata*

Calcium-dependent protein kinase (CPK), a type of calmodulin protein, is predominantly found in plants and protists, playing a crucial role in response to various biological and abiotic stresses (Harmon et al., 2000; Zhang et al., 2015; Zuo et al., 2024). In this study, we identified a total of 32 *CPK* genes family members in *H. subcapitata*, and phylogenetic analysis revealed that *HsCPKs* can be categorized into four distinct subfamilies (**Figure 1a**). The results showed that the conserved motifs of the 32 *HsCPKs* were relatively similar (**Figure 2b**). Furthermore, motif structures within the same subfamily showed greater similarity, particularly among genes situated on adjacent branches. For instance, the motif structures of the 11 *HsCPK* members within subfamily II were similar, especially the sister group of *HsCPK24* and *HsCPK26*. This similarity was also consistent with observations in *CPK* research of *Boea hygrometrica* (Shi et al., 2022). However, members of subfamilies I and IV displayed slight variability in their motifs. For example, *HsCPK6* and *HsCPK22* in subfamily I lacked motif 4 and motif 7 at the N-terminal region. According to a previous study, variations in motifs may be contributed to functional differentiation among gene family members (An et al., 2024). In summary, while specific variations existed, the motif structure of the *CPK* gene family was highly conserved in *H. subcapitata*.

### 4.2 Gene structure variability of *CPK* gene family in *H. subcapitata*

Genes within the same subfamily demonstrated relatively comparable exon-intron structures as well as intron phases (**Figure 2a**). The number of introns in the 32 *HsCPK* genes ranges from 7 to 12. Among these, members of subfamily I contained the highest number of introns (11-12), while those in subfamily IV contained the fewest (only 7). Furthermore, gene structure of subfamily I differed significantly from those of the other three subfamilies. This suggests that members of subfamily I may represent the most ancient members of the *CPK* gene family. Zhang and Choi (2001) proposed that *CPK* gene introns possess an ancient origin, with genes undergoing intron-based recombination to generate novel exon combinations. This phenomenon aligned also closely with observed gene structures across all four *CPK* gene subfamilies in *A. thaliana* (Zhang and Choi, 2001). Consequently, intron abundance may correlate with gene antiquity, which underscoring the *CPK* gene structure variability across plant species.

In this study, the *HsCPK* gene was found to be located on 14 chromosomes, with a concentration on chromosomes 1 and 2 (**Figure 3**). The results of collinearity analysis revealed 10 pairs of collinear genes, all generated by segmental duplication (**Supplementary Table S4**). These genes pairs were distributed across all four subfamilies: three pairs of genes replicated in subfamily IV; two pairs in subfamily III; four pairs in subfamily II; only one pair in subfamily I (**Supplementary Table S4**). Plenty of studies suggested the expansion of *CPK* gene families can be attributed to the segmental duplication (Xu et al., 2012; Zuo et al., 2013). For example, there were two tandem replications and 12 segmental duplications happened in *CPK* gene family of *Populus trichocarpa* (Zuo et al., 2013); 8 segmental duplication events of *CPK* gene family in *Gossypium raimondii* (Liu et al., 2014); 7 segmental duplications displayed in *CPK* genes family of Rice (Asano et al., 2005). Therefore, we deemed that segmental duplication significantly contributes to the expansion of the *CPK* gene family of *H. subcapitata*, and segmental duplication is the primary evolutionary mechanism for *CPKs* expansion.

### 4.3 The role of *CPK* gene family in karst adaption of *H. subcapitata*

The core stresses in karst habitats are drought and high calcium (Ford and Williams, 2007), and cis-acting element analysis indicated that the elements in the *CPK* family exhibited obviously functional adaptability to drought and high calcium stresses. Our results showed that the MeJA-responsive elements and ABA-responsive elements were widely enriched in each subfamily of the *HsCPK* family (**Figure 4**). Previous studies have confirmed that *A. thaliana* activates the MeJA and ABA signaling pathways to synergistically regulate cellular calcium homeostasis and drought stress metabolism (Khan et al., 2023). Especially, ABA plays a significant role in calcium signaling pathways in plants (Zhu, 2016). Meanwhile, the defense and stress response elements specific to subfamily II and the drought-inducible MYB sites enriched in subfamily IV further enhanced the adaptability of the *HsCPK* family to the combined drought-high calcium stress in karst habitats. In addition, the low-temperature responsive elements enriched in Subfamily III were not direct response elements to the core stresses in karst habitats. However, they still reflected the comprehensive adaptability of the *HsCPK* family to habitat stresses. In summary, cis-acting element analysis confirmed that this *HsCPK* family was key to adapt in drought-high calcium habitats.

Selection pressure analysis showed that both subfamilies III and IV experienced strong positive selection, and subfamilies I and II were under neutral selection. Our results also showed that *CPK* was expressed in root, stem and leaf of *H. subcapitata* (**Figure 5**). Notably, Calcium stress experiments demonstrated elevated expression levels of the *CPK* gene across root, stem, and leaf, with the most pronounced increase observed in root under 10 mmol/L CaCl_2_ treatment (**Figure 6**). For instance, *HsCPK13*, *HsCPK20*, *HsCPK25*, and *HsCPK32* exhibited high expression in root, *HsCPK20* and *HsCPK31* were highly expressed in stem, while *HsCPK8* and *HsCPK28* showed elevated expression in leaf. These RT-qPCR results were validated to confirm consistency with the heatmap (**Figure 5a**). Furthermore, *HsCPK13*, *HsCPK20*, and *HsCPK28* exhibited significant up-regulation in stem, while *HsCPK31* and *HsCPK32* showed marked increases in leaf. Previous studies suggested that the *CPK* genes were benefit to the plants in karst regions. For instance, the expression of *CPK* gene in *Medicago lupulina* also showed an increasing trend in karst areas (Zhang et al., 2019). In addition, *CPKs* are extensively involved in plant adaptation to environmental stresses such as drought and high salinity. For example, *OsCDPK29* in rice responds to multiple stresses and can be induced by drought, cold, and salt stress (Wan et al., 2007); *TaCDPK13* was involved in the ROS signaling pathway in wheat in response to environmental drought stress (Hu et al., 2020). *H. subcapitata* mainly distributes in Asian karst regions (Li and Wang, 2005; Wen et al., 2010), and faces the calcium-rich environment and drought threat. The expansion of *CPK* gene family plays an important role in the adaptation of *H. subcapitata* to the karst environment. These results collectively revealed the function of *CPK* genes for plants to cope with complex environmental stresses.

## 5 Conclusions

In this study, the identified 32 members of *CPK* gene family exhibited high conservatism in *H. subcapitata*, and they were distributed on 14 chromosomes. Subcellular localization analysis revealed that *HsCPKs* were mainly localized in chloroplasts and cytoplasm. Ten gene pairs have resulted from fragment replication. The 32 *HsCPKs* were classified into four subfamilies, with 11 members in subfamily II and IV respectively. Furthermore, the *CPK* gene family in *H. subcapitata* underwent intron loss during evolution. Subfamilies III and IV exhibited strong positive selection. Notably, the expression levels of *CPK* gene family were high in the three vegetative tissues of *H. subcapitata* under calcium stress. The cis-acting element analysis indicated that *CPK* genes possessed key calcium stress response elements and drought-inducible sites. This study provides a foundational understanding for further investigations into the roles of the *CPK* gene family within Gesneriaceae.

## Data Availability

The datasets presented in this study can be found in online repositories. The names of the repository/repositories and accession number(s) can be found below: https://ngdc.cncb.ac.cn, CRA029625 https://ngdc.cncb.ac.cn, GWHFICV00000000.

## Supplementary material

Supplementary Figure 1 The motif sequence in *HsCPK* gene family.

Supplementary Table 1 The development of *H. subcapitata* under different concentrations of CaCl_2_ treatment.

Supplementary Table 2 Primer information used for RT-qPCR.

Supplementary Table 3 Basic information of the *CPK* gene family in *H. subcapitata.*

Supplementary Table 4 Collinearity of the *CPK* gene family in *H. subcapitata.*
