# Supplementary material for: Genome-wide identification of the CPK gene family and associated responses to calcium stress in Hemiboea subcapitata
Source: Front Plant Sci. 2026 Jan 28;17:1745553. doi: 10.3389/fpls.2026.1745553 (PMC12891223; doi:10.3389/fpls.2026.1745553)
Supplement: Supplementary Figure 1 — The motif sequence in HsCPK gene family. [file DataSheet1.pdf]

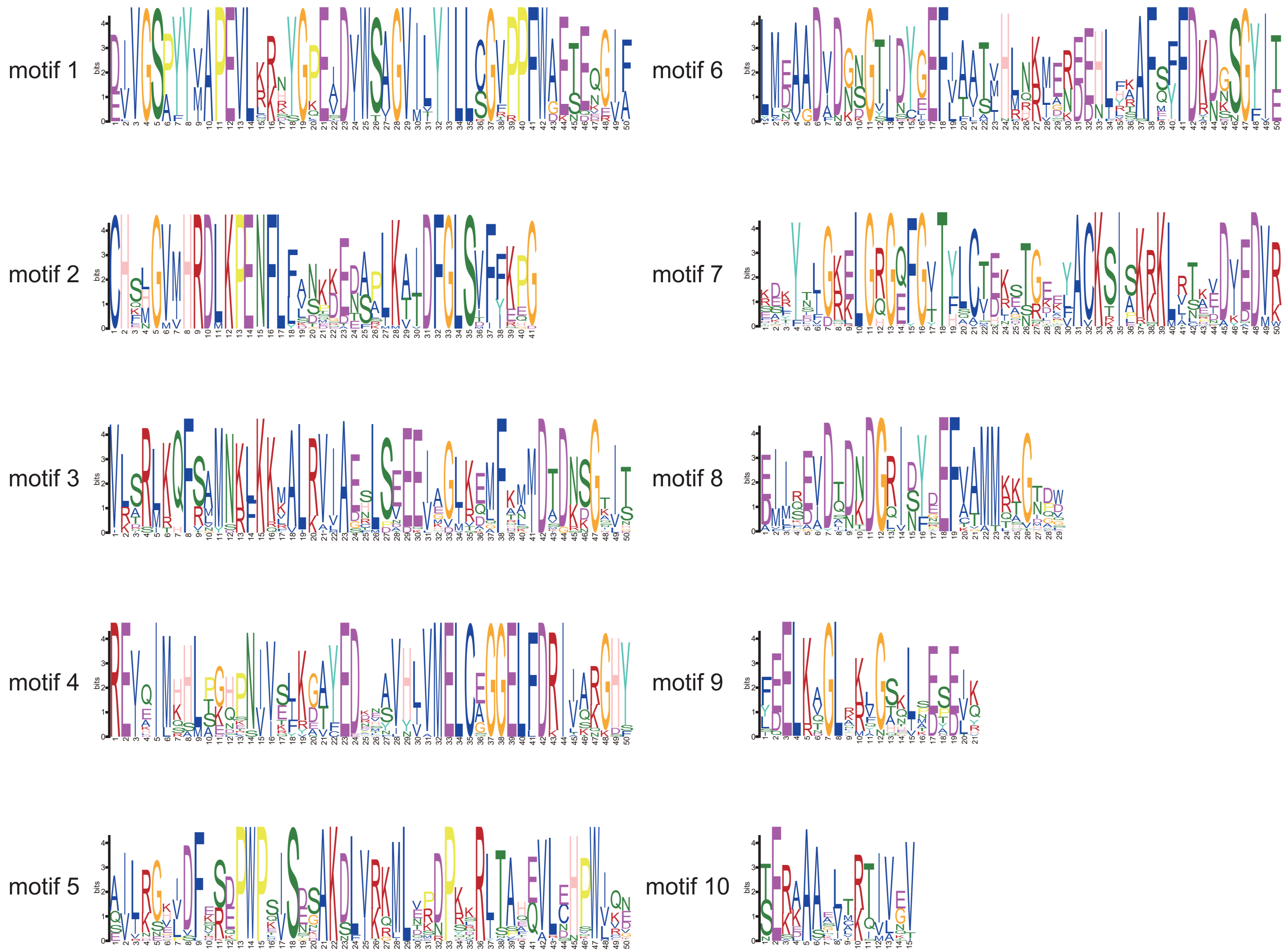

Supplementary Figure S1. The motif sequence in *HsCPK* gene family. The length of the amino acid was inferred by ruler at bottom. Different colors of letters represent different kinds of amino acids residues, and the size of letters represents the frequency of amino acid occurrence.
