# Supplementary material for: Genome-wide identification of the CPK gene family and associated responses to calcium stress in Hemiboea subcapitata
Source: Front Plant Sci. 2026 Jan 28;17:1745553. doi: 10.3389/fpls.2026.1745553 (PMC12891223; doi:10.3389/fpls.2026.1745553)
Supplement: Supplementary Table 1 — The development of H. subcapitata under different concentrations of CaCl2 treatment. [file Table1.docx]

**Supplementary Table S1. The development of *H. subcapitata* under different concentrations of CaCl_2_ treatment**

| Ca²⁺ concentration (mmol/L) | Before treatment (cm) | | | | | After treatment (cm) | | | | |
| --- | --- | --- | --- | --- | --- | --- | --- | --- | --- | --- |
|  | Root length | Stem length | Leaf  length | Leaf  width | Plant height | Root length | Stem length | Leaf  length | Leaf  width | Plant height |
| 0 mmol/L | 8.6 | 9.6 | 6.5 | 5.6 | 17.4 | 9.2 | 9.3 | 7.2 | 5.5 | 18.1 |
|  | 6 | 11.9 | 5.4 | 3.7 | 17.4 | 6.3 | 9.7 | 5.8 | 4.1 | 17.9 |
|  | 6.1 | 8.5 | 5.8 | 5 | 17.5 | 6.4 | 7.8 | 6.2 | 4.1 | 16.9 |
| 5 mmol/L | 6.9 | 7.4 | 5.7 | 4.1 | 15.3 | 6.2 | 6.6 | 5.7 | 4.3 | 16.2 |
|  | 6.1 | 8.3 | 7.3 | 5.7 | 16.1 | 6.6 | 9.3 | 7.8 | 5.6 | 19.3 |
|  | 6.5 | 7.4 | 6.5 | 4.7 | 16.9 | 6.4 | 8 | 6.8 | 4.6 | 17.9 |
| 10 mmol/L | 6.4 | 9.5 | 7.7 | 5.8 | 17.9 | 6.7 | 8.9 | 8.4 | 5.6 | 17.1 |
|  | 7.3 | 9.5 | 5.6 | 4.2 | 17.1 | 7.9 | 10.1 | 5.5 | 4.1 | 18.5 |
|  | 4.3 | 9.1 | 7.5 | 5.9 | 18.5 | 6.5 | 9.9 | 8.2 | 6.1 | 19.8 |
| 20 mmol/L | 5.7 | 8.2 | 6.5 | 4.9 | 14.8 | 6.5 | 8.4 | 6 | 4.8 | 16.9 |
|  | 3.2 | 7.8 | 5.5 | 4.8 | 15.3 | 3.6 | 9 | 5.3 | 4 | 14.9 |
|  | 5.3 | 10.2 | 5.8 | 4.3 | 16.5 | 5.5 | 8.5 | 4.8 | 4 | 14.2 |
