# Supplementary material for: Genome-wide identification of the CPK gene family and associated responses to calcium stress in Hemiboea subcapitata
Source: Front Plant Sci. 2026 Jan 28;17:1745553. doi: 10.3389/fpls.2026.1745553 (PMC12891223; doi:10.3389/fpls.2026.1745553)
Supplement: Supplementary Table 2 — Primer information used for RT-qPCR. [file Table2.docx]

**Supplementary Table S2. Primer information used for RT-qPCR**

| Gene | Primer | Length(bp) | GC content | Tm (℃) |
| --- | --- | --- | --- | --- |
| β-actin-F | GCTAGTGGCCGTACAACTG | 173 | 57.9 | 56.2 |
| β-actin-R | GAACATATACCCTCTTTCAG | 173 | 40 | 46.6 |
| *CPK8*-F | CCAATACCGAGGAAGTTATCAG | 154 | 45.5 | 52.4 |
| *CPK8*-R | AGACTGTTGAATCTCTCACGAG | 154 | 45.5 | 53.8 |
| *CPK13*-F | ATTGAACGGGATAGCAGGG | 168 | 52.6 | 54.6 |
| *CPK13*-R | AGCATTCTCGTCCTCACACG | 168 | 55 | 57.5 |
| *CPK20*-F | GAGAAAGATGGGAAACGACG | 179 | 50 | 53.4 |
| *CPK20*-R | TGACCTTGTCCGTGTCTACG | 179 | 55 | 56.9 |
| *CPK25*-F | ACCATCTCACTGGAAACCG | 223 | 52.6 | 54.7 |
| *CPK25*-R | GGTCACAATCTGCCTACACAG | 223 | 52.4 | 55.8 |
| *CPK28*-F | TCTGGCTGGTCACAAGAAC | 180 | 52.6 | 54.9 |
| *CPK28*-R | GCCTCCACAACTCCTACAAC | 180 | 55 | 55.9 |
| *CPK31*-F | GCTGGTGCCAATCTAAATG | 223 | 47.4 | 51.3 |
| *CPK31*-R | ACCCATACTCCTGACAAACG | 223 | 50 | 54.4 |
| *CPK32*-F | CGAGTAAGAAGGAGAATGCTGC | 203 | 50 | 55.7 |
| *CPK32*-R | AAATGGAGGAACGCCACAC | 203 | 52.6 | 55.8 |
