# Supplementary material for: Genome-wide identification of the CPK gene family and associated responses to calcium stress in Hemiboea subcapitata
Source: Front Plant Sci. 2026 Jan 28;17:1745553. doi: 10.3389/fpls.2026.1745553 (PMC12891223; doi:10.3389/fpls.2026.1745553)
Supplement: Supplementary Table 3 — Basic information of the CPK gene family in H. subcapitata. [file Table3.docx]

**Supplementary Table S3. Basic information of the *CPK* gene family in *H. subcapitata***

| Gene name | Gene ID | Chromosome | Chain | Start point | End point | Subcellular localization |
| --- | --- | --- | --- | --- | --- | --- |
| *HsCPK1* | LG1.g01760 | Chr1 | - | 36260053 | 36265548 | Chloroplast |
| *HsCPK2* | LG1.g01792 | Chr1 | + | 36555799 | 36558703 | Chloroplast |
| *HsCPK3* | LG1.g02070 | Chr1 | + | 39390803 | 39395530 | Cytoplasm |
| *HsCPK4* | LG1.g02460 | Chr1 | + | 44320377 | 44322587 | Cytoplasm |
| *HsCPK5* | LG1.g02484 | Chr1 | + | 44598761 | 44602314 | Cytoplasm |
| *HsCPK6* | LG2.g03471 | Chr2 | + | 2756281 | 2758577 | Cytoplasm |
| *HsCPK7* | LG2.g04047 | Chr2 | + | 9362676 | 9364894 | Endoplasmic reticulum |
| *HsCPK8* | LG2.g04067 | Chr2 | + | 9601777 | 9606916 | Plasma membrane |
| *HsCPK9* | LG2.g05197 | Chr2 | + | 49687854 | 49691646 | Chloroplast |
| *HsCPK10* | LG2.g05346 | Chr2 | - | 51653986 | 51660345 | Chloroplast |
| *HsCPK11* | LG3.g06194 | Chr3 | + | 4990195 | 4994169 | Plasma membrane |
| *HsCPK12* | LG3.g06468 | Chr3 | - | 7603423 | 7606695 | Chloroplast |
| *HsCPK13* | LG3.g06896 | Chr3 | - | 12698394 | 12703806 | Chloroplast |
| *HsCPK14* | LG3.g07610 | Chr3 | - | 24076962 | 24081043 | Chloroplast |
| *HsCPK15* | LG4.g09903 | Chr4 | - | 38628282 | 38632156 | Cytoplasm |
| *HsCPK16* | LG4.g10236 | Chr4 | + | 43960130 | 43963886 | Cytoplasm |
| *HsCPK17* | LG4.g10377 | Chr4 | - | 45495850 | 45501718 | Chloroplast |
| *HsCPK18* | LG5.g11704 | Chr5 | - | 16038428 | 16049507 | Nucleus |
| *HsCPK19* | LG6.g13058 | Chr6 | + | 13428408 | 13434886 | Chloroplast |
| *HsCPK20* | LG6.g13682 | Chr6 | + | 38987800 | 38991945 | Chloroplast |
| *HsCPK21* | LG7.g14413 | Chr7 | - | 1819591 | 1825241 | Chloroplast |
| *HsCPK22* | LG7.g15044 | Chr7 | - | 1819591 | 1825241 | Cytoplasm |
| *HsCPK23* | LG7.g15477 | Chr7 | - | 38354297 | 38364604 | Chloroplast |
| *HsCPK24* | LG8.g16038 | Chr8 | - | 1680478 | 1684576 | Mitochondria |
| *HsCPK25* | LG8.g16640 | Chr8 | - | 9487883 | 9492617 | Mitochondria |
| *HsCPK26* | LG9.g17755 | Chr9 | - | 1250916 | 1254785 | Cytoplasm |
| *HsCPK27* | LG9.g18811 | Chr9 | + | 16008336 | 16011347 | Nucleus |
| *HsCPK28* | LG10.g20682 | Chr10 | - | 36551932 | 36556716 | Nucleus |
| *HsCPK29* | LG12.g23341 | Chr12 | + | 3776148 | 3781035 | Nucleus |
| *HsCPK30* | LG13.g25046 | Chr13 | - | 3429867 | 3437693 | Chloroplast |
| *HsCPK31* | LG14.g27137 | Chr14 | - | 26951372 | 26967626 | Chloroplast |
| *HsCPK32* | LG16.g29391 | Chr16 | + | 26192392 | 26197170 | Cytoplasm |
