# Supplementary material for: Genome-wide identification of the CPK gene family and associated responses to calcium stress in Hemiboea subcapitata
Source: Front Plant Sci. 2026 Jan 28;17:1745553. doi: 10.3389/fpls.2026.1745553 (PMC12891223; doi:10.3389/fpls.2026.1745553)
Supplement: Supplementary Table 4 — Collinearity of the CPK gene family in H. subcapitata. [file Table4.docx]

**Supplementary Table S4. Collinearity of the *CPK* gene family in *H. subcapitata***

| Gene pair | Chromosome | Collinear gene 1 | | Chromosome | Collinear gene 2 | |
| --- | --- | --- | --- | --- | --- | --- |
|  |  | Gene ID | Gene name |  | Gene ID | Gene name |
| 1 | Chr1 | LG1.g02460 | *HsCPK4* | Chr2 | LG2.g04047 | *HsCPK7* |
| 2 | Chr10 | LG10.g20682 | *HsCPK28* | Chr12 | LG12.g23341 | *HsCPK29* |
| 3 | Chr4 | LG4.g10377 | *HsCPK17* | Chr5 | LG5.g11704 | *HsCPK18* |
| 4 | Chr2 | LG2.g05346 | *HsCPK10* | Chr7 | LG7.g15477 | *HsCPK23* |
| 5 | Chr1 | LG1.g02484 | *HsCPK5* | Chr2 | LG2.g04067 | *HsCPK8* |
| 6 | Chr1 | LG1.g01792 | *HsCPK2* | Chr2 | LG2.g03471 | *HsCPK6* |
| 7 | Chr16 | LG16.g29391 | *HsCPK32* | Chr4 | LG4.g09903 | *HsCPK15* |
| 8 | Chr13 | LG13.g25046 | *HsCPK30* | Chr14 | LG14.g27137 | *HsCPK31* |
| 9 | Chr3 | LG3.g07610 | *HsCPK14* | Chr6 | LG6.g13682 | *HsCPK20* |
| 10 | Chr8 | LG8.g16038 | *HsCPK24* | Chr9 | LG9.g17755 | *HsCPK26* |
